# Assessment of Potential Risks of Dietary RNAi to a Soil Micro-arthropod, *Sinella curviseta* Brook (Collembola: Entomobryidae)

**DOI:** 10.3389/fpls.2016.01028

**Published:** 2016-07-15

**Authors:** Huipeng Pan, Linghua Xu, Jeffrey E. Noland, Hu Li, Blair D. Siegfried, Xuguo Zhou

**Affiliations:** ^1^Department of Entomology, University of Kentucky, LexingtonKY, USA; ^2^Department of Entomology and Nematology, University of Florida, GainesvilleFL, USA

**Keywords:** RNA interference, gene cloning, mRNA expression, life history, *Sinella curviseta*, risk assessment

## Abstract

RNAi-based genetically engineered (GE) crops for the management of insect pests are likely to be commercialized by the end of this decade. Without a workable framework for conducting the ecological risk assessment (ERA) and a standardized ERA protocol, however, the utility of RNAi transgenic crops in pest management remains uncertain. The overall goal of this study is to assess the risks of RNAi-based GE crops on a non-target soil micro-arthropod, *Sinella curviseta*, which could be exposed to plant-protected dsRNAs deposited in crop residues. Based on the preliminary research, we hypothesized that insecticidal dsRNAs targeting at the western corn rootworm, *Diabrotica virgifera virgifera*, a billion-dollar insect pest, has no adverse impacts on *S. curviseta*, a soil decomposer. Following a tiered approach, we tested this risk hypothesis using a well-designed dietary RNAi toxicity assay. To create the worst-case scenario, the full-length cDNA of *v-ATPase* subunit *A* from *S. curviseta* were cloned and a 400 bp fragment representing the highest sequence similarity between target pest and non-target arthropods was selected as the template to synthesize insecticidal dsRNAs. Specifically, 10-days-old *S. curviseta* larvae were subjected to artificial diets containing *v-ATPase A* dsRNAs from both *D. v. virgifera* (dsDVV) and *S. curviseta* (dsSC), respectively, a dsRNA control, β-*glucuronidase*, from plant (dsGUS), and a vehicle control, H_2_O. The endpoint measurements included gene expression profiles, survival, and life history traits, such as developmental time, fecundity, hatching rate, and body length. Although, *S. curviseta* larvae developed significantly faster under the treatments of dsDVV and dsSC than the vehicle control, the combined results from both temporal RNAi effect study and dietary RNAi toxicity assay support the risk hypothesis, suggesting that the impacts of ingested arthropod-active dsRNAs on this representative soil decomposer are negligible.

## Introduction

RNA interference (RNAi) is an evolutionarily conserved mechanism that relies on the production of short interfering RNAs (siRNAs; 20–30 nucleotides in length), which promote degradation or translation repression of homologous mRNAs. The ability to manipulate the expression of specific genes in insects provides an important functional genomics tool and has laid the foundation for the development of environmentally friendly pest management approaches (Burand and Hunter, 2013). RNAi-based gene regulation has been reported in several insect orders with tremendous variability, including Diptera (Galiana-Arnoux et al., 2006; Wang et al., 2006; Whyard et al., 2009; Coy et al., 2012), Coleoptera (Baum et al., 2007; Zhu et al., 2011; Xiao et al., 2015), Hemiptera (Zha et al., 2011; Bansal and Michel, 2013; Xu H.J. et al., 2015), Hymenoptera (Yoshiyama et al., 2013), Lepidoptera (Turner et al., 2006; Guo Z. et al., 2015), Thysanoptera (Badillo-Vargas et al., 2015), Orthoptera (Guo Y. et al., 2015), Isoptera (Zhou et al., 2006, 2008), and Blattodea (Martín et al., 2006), which makes it possible to develop RNAi technology for the control of a variety of insect pests (Gordon and Waterhouse, 2007; Huvenne and Smagghe, 2010; Swevers and Smagghe, 2012).

The western corn rootworm, *Diabrotica virgifera virgifera* LeConte (Coleoptera: Chrysomelidae), has been a serious maize pest in the US since 1940s following initial expansion from isolated regions of the western plain states, Kansas and Colorado (Gray et al., 2009). Spread from these localized populations was likely due to continuous planting of maize and the development of resistance to synthetic insecticides, which facilitated the subsequent invasion into Midwestern states from the mid-1950 to 1970s and as far as Virginia by the 1980s (Levine and Oloumi-Sadeghi, 1991). Crop losses and management costs for *D. v. virgifera* in the US are reported to exceed $1 billion annually (Gray et al., 2009). This problem, however, is not isolated to the US alone. In 1992, *D. v. virgifera* was identified in Serbia, Yugoslavia, likely due to international travels between the US and Europe (Gray et al., 2009). Since then, *D. v. virgifera* has been found in 20 European countries (Miller et al., 2005; Gray et al., 2009). Rootworm controls have been seriously challenged by the insect’s ability to develop resistance to agricultural practices (behavioral resistance to crop rotation), chemical controls (resistance to synthetic insecticides), and, recently, genetically engineered (GE) maize expressing *Bacillus thuringiensis* Cry toxins (resistance to Cry3Bb1 and mCry3A; Levine and Oloumi-Sadeghi, 1991; Gray et al., 2009; Gassmann et al., 2014).

The first *B. thuringiensis* maize to control *D. v. virgifera* was introduced onto the market in 2003, and by 2009 this *B. thuringiensis* trait constituted nearly half of all maize planted in the US (James, 2009). With the rapid adoption of this GE maize variety, coupled with the lack of compliance by farmers (e.g., limited or no refuges), resistance to Cry3Bb1, a *B. thuringiensis* toxin specific to rootworms, was quickly developed in the field (Gassmann et al., 2011). A subsequent study showed that these *D. v. virgifera* populations were cross-resistant to a modified *B. thuringiensis* toxin, mCry3A, which led to severe injury to *B. thuringiensis* maize in the field (Gassmann et al., 2014). To counter the remarkable adaptability of rootworms, emerging biotechnologies with a brand new mode of action (MOA) are needed for the long-term, sustainable management of this insect pest.

RNAi-based transgenic traits offer a paradigm-shifting biotechnology and complement the existing management practices with a completely different MOA. *In planta* RNAi, delivering dsRNA through transgenic plants, has been pioneered in several insect pest species, including western corn rootworm, *D. v. virgifera* (Baum et al., 2007),Colorado potato beetle, *Leptinotarsa decemlineata* (Zhang et al., 2015), green peach aphid, *Myzus persicae* (Pitino et al., 2011; Mao and Zeng, 2014), cotton bollworm, *Helicoverpa armigera* (Mao et al., 2007, 2011, 2013), tobacco hornworm, *Manduca sexta* (Kumar et al., 2012), brown planthopper, *Nilaparvata lugens* (Zha et al., 2011), and English grain aphid, *Sitobion avenae* (Xu et al., 2014). Baum et al. (2007) initially developed a transgenic trait expressing *D. v. virgifera vacuolar ATPase subunit A*. By suppressing the translation of *D. v. virgifera v-ATPase*, this GE maize caused severe larval mortality and stunted growth, which resulted in significantly less root damage relative to empty-vector and control plants (Baum et al., 2007). Most recently, a GE event, MON 87411, which stacks one herbicide tolerance trait with two insect resistance traits, has been deregulated by the USDA’s Animal and Plant Health Inspection Service (APHIS). One of the traits designed to control *D. v. virgifera* involves a suppression cassette that targets *D. v. virgifera Snf7* gene (*DvSnf7*). Upon consumption, the plant-produced dsRNA in MON 87411 is recognized by *D. v. virgifera* RNAi machinery. The subsequent suppression of *DvSnf7*, a housekeeping gene and an essential component of cellular machinery known as endosomal sorting complex required for transportation, leads to *D. v. virgifera* mortality (Bolognesi et al., 2012). As of today, the determination of non-regulated status of MON 87411 is in the process at the US Environmental Protection Agency (EPA), and the US Food and Drug Administration (FDA). Although, technical difficulties and regulatory concerns still exist (Lundgren and Duan, 2013; Casacuberta et al., 2015; Roberts et al., 2015; Xu L.H. et al., 2015), RNAi-based pest controls are likely to be commercialized by the end of this decade (Kupferschmidt, 2013).

Prior to the commercial release of RNAi crops, a risk assessment framework to evaluate the effects on non-target arthropods must be established (Romeis et al., 2008; Lundgren and Duan, 2013; USEPA, 2013, 2014; EFSA, 2014; Casacuberta et al., 2015; Roberts et al., 2015; Xu L.H. et al., 2015). In previous environmental risk assessments of transgenic *B. thuringiensis* crops, collembolans represent a class of soil-dwelling micro-arthropods used to test the effect of GE products in the soil ecosystem. Collembola (springtails) represents cosmopolitan micro-arthropods that inhabit nearly all soil types and plays important roles as decomposers of leaf litter and soil organic matter (Hopkin, 1997; Cole et al., 2001). *Sinella curviseta* Brook (Collembola: Entomobryidae) and *Folsomia candida* (Collembola: Isotomidae), often colonize similar habitats, are the two global representatives for soil-dwelling animals (ISO, 1999; Xu et al., 2009). *F. candida* has been used extensively for *B. thuringiensis* crop risk assessment (Yu et al., 1997; Al-Deeb et al., 2003; Clark and Coats, 2006; Bai et al., 2010, 2011; Römbke et al., 2010; Bakonyi et al., 2011; Yuan et al., 2013; Yang et al., 2015), and *S. curviseta* is listed, among others, as an alternative species in the Organization for Economic Cooperation and Development (OECD) guideline (Bandow et al., 2014a,b). To date, there is no information on the susceptibility of collembolans to RNAi. Given that the newly developed RNAi maize is primarily targeting at a soil-dwelling insect pest which shares the same habitat with collembolans, it is germane to investigate the impact of *D. v. virgifera* active dsRNAs on a representative soil decomposer, *S. curviseta*.

Here, following a tiered approach, we examined the risk hypothesis that *D. v. virgifera* active *v-ATPase A* dsRNA has no adverse impact on the non-target *S. curviseta*. As a Tier I assessment, the worst case scenario was established, which involves exposure to a maximum hazard dose with purified active ingredients in artificial diets (USEPA, 1998). To test this risk hypothesis under the worst case scenario, we (1) cloned and sequenced *v-ATPase A* gene from *S. curviseta* and selected a cDNA fragment representing the highest sequence similarity between target and non-target insects; (2) developed a dietary RNAi toxicity assay; and (3) assessed the impacts of ingested dsRNAs on the gene expression and life history traits (i.e., survival rate, fecundity, hatching rate, and body length) of *S. curviseta*.

## Results

### Cloning of *v-ATPase subunit A*

**FIGURE 1:**
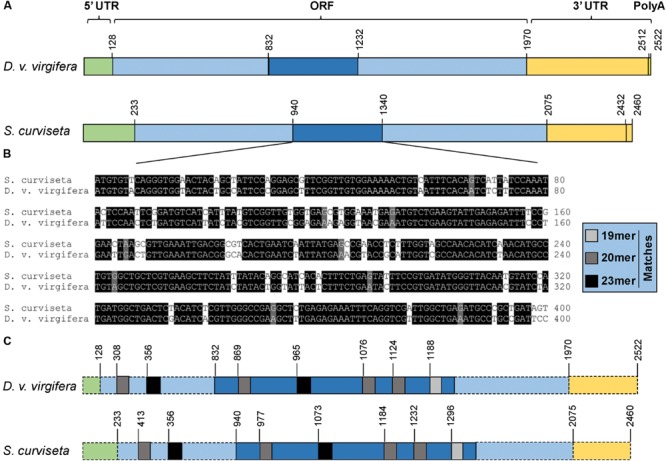
**Schematic comparison of *v-ATPase A* from targeted insect pest, *Diabrotica virgifera* and non-target *Sinella curviseta.* (A)** Schematic drawing of the primary structures of *v-ATPase A* cDNA from *S. curviseta* and *D. v. virgifera*. **(B)** The alignment of a highly conserved region within the ORFs of *v-ATPase A* from *S. curviseta* and *D. v. virgifera*. This 400 bp fragment, which has the highest sequence similarity among all the tested surrogate species, was selected as the target template to synthesis insecticidal dsRNAs. **(C)** This schematic drawing shows the distribution and number of identical sequences, ranging from 19 to 23-mer matches, imbedded in the ORFs of *v-ATPase A* from *S. curviseta* and *D. v. virgifera*.

Degenerate primers and touchdown polymerase chain reaction (PCR) were used to amplify the first fragment of *v-ATPase A* from *S. curviseta*. The entire coding region was obtained by a combination of RT-PCR (reverse transcription polymerase chain reaction) and RACE (rapid amplification of cDNA ends). The complete cDNA sequence of *S. curviseta v-ATPase* has 2460 bp, including a 232 bp 5′-untranslated region, a 383 bp 3′-untranslated region, and an open reading frame (ORF) of 1845 bp that encodes a protein of 614 amino acids (**Figure 1A**). In comparison, *D. v. virgifera v-ATPase A* cDNA is 2522 bp in length, including a 127 bp 5′-untranslated region, a 553 bp 3′-untranslated region, and a1842 bp ORF that encodes a protein of 613 amino acids (**Figure 1A**). In addition, pair-wise comparison showed that these two ORFs share a 75.0% nucleotide sequence similarity (**Supplementary Figure S1**). The 400 bp region with the highest sequence similarity (85%) was selected as the template to synthesize arthropod-active dsRNAs (**Figure 1B**).

### N-mer Matches

The ORF of *S. curviseta* and *D. v. vugifera* shares 19 19-, 12 20-, six 21-, four 22-, and two 23-nt contiguous matches (**Figure 1C**, Supplementary Table S2). The conserved 400 bp region shares 12 19-, seven 20-, three 21-, two 22-, and one 23-nt contiguous matches (**Figure 1C**, Supplementary Table S2, highlighted in gray).

### Phylogenetic Analysis

**FIGURE 2:**
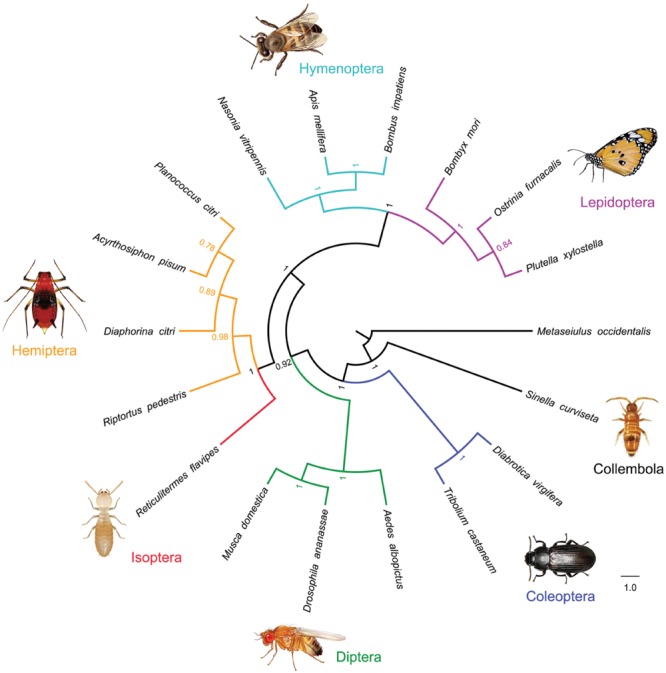
**Phylogenetic analysis of *v-ATPase A* in insects.** Amino acid sequences were aligned using the MAFFT algorithm in the TranslatorX online platform. Alignments were then checked and corrected manually in MEGA v6.0. The WAG+G model was the best-fit amino acid substitution model selected by ProtTest 2.4 under AIC criteria. MrBayes v3.2.3 with WAG+G model was used for the phylogenetic analysis.

Phylogenetic analysis supports the sister relationship between *S. curviseta* (Collembola) and other insects (Insecta) based on *v-ATPase A* amino acid sequence using Bayesian analysis (PP = 1.0; **Figure 2**).

### dsRNA Stability Assay

**FIGURE 3:**
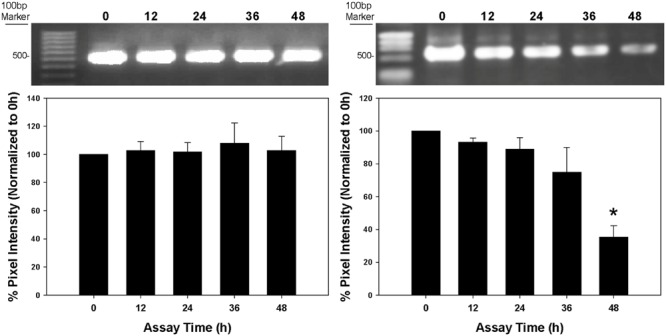
**dsRNA stability in the presence and absence of *S. curviseta*. Left**: without *S. curviseta, v-ATPase A* dsRNA incorporated into the diets was stable throughout 2-days assay period [H(4) = 7.5462, *P =* –0.109]. **Right:** in the presence of *S. curviseta*, however, *v-ATPase A* dsRNA started to degrade at the end of the 2-days assay [H(4) = 14.230, *P* = 0.007]. Values shown are percentages (% pixel intensity of the *v-ATPase A* dsRNA gel band) ± SE. Asterisk (^∗^) means *v-ATPase A* dsRNAs was stable for the first 36 h in the presence of *S. curiviseta*, while it started to degrade by 48 h.

Percent (%) pixel intensity of the expected gel band was measured every 12 h across two assay days and normalized to 0 h time point. For diet without *S. curiviseta*, no significant degradation of *v-ATPase A* dsRNAs was observed [H(4) = 7.5462, *P* = 0.109, **Figure 3**]. Similarly, *v-ATPase A* dsRNAs was stable for the first 36 h in the presence of *S. curiviseta*, while it started to degrade by 48 h [H(4) = 14.230, *P* = 0.007, **Figure 3**]. This, however, should not impact the outcome because the diet was replaced every other day (at 48 h) for the duration of dietary RNAi toxicity assay.

### Temporal Profile of Dietary RNAi in *S. curviseta*

**FIGURE 4:**
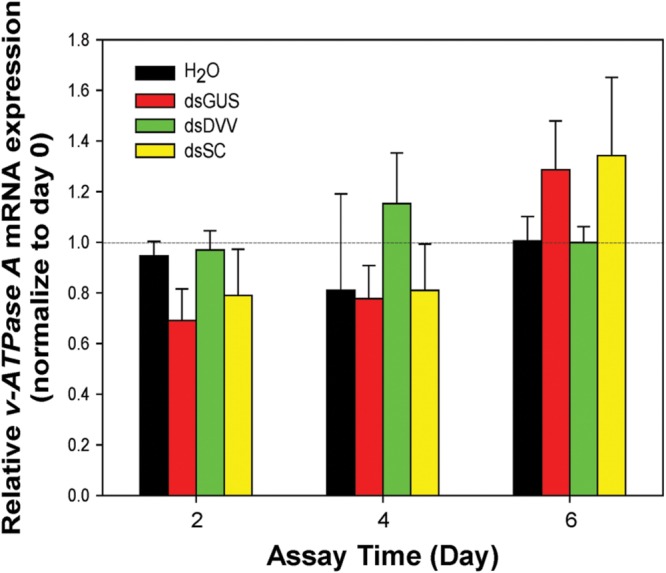
**Temporal profile of *v-ATPase A* expression in *S. curviseta*.** The expression profile of *S. curviseta v-ATPase A* was documented on days 2, 4, and 6. The relative expression of *v-ATPase A* transcripts was normalized to a reference gene *28S rRNA*, and the transcription level of *v-ATPase A* in the 10-days-old untreated larvae were set to 1. See text for treatment details. Values are mean ± SE.

The linear regression equation, correlation coefficient and PCR efficiency for the standard curve are shown in **Supplementary Figure S2**. The expression profile of *28S rRNA* across all experimental conditions was documented in **Supplementary Figure S3** using Ct values extracted from the original qRT-PCR dataset. *28S rRNA* was stably expressed throughout the entire experiment, and therefore, used as a reference to normalize the target gene expression. *v-ATPase A* expression was not affected by the treatment (*F*_3,24_ = 0.276, *P* = 0.842), time (*F*_2,24_ = 0.063, *P* = 3.100) and interactions between these two factors (*F*_6,24_ = 0.982, *P* = 0.459; **Figure 4**).

### Dietary RNAi Toxicity Assay

**FIGURE 5:**
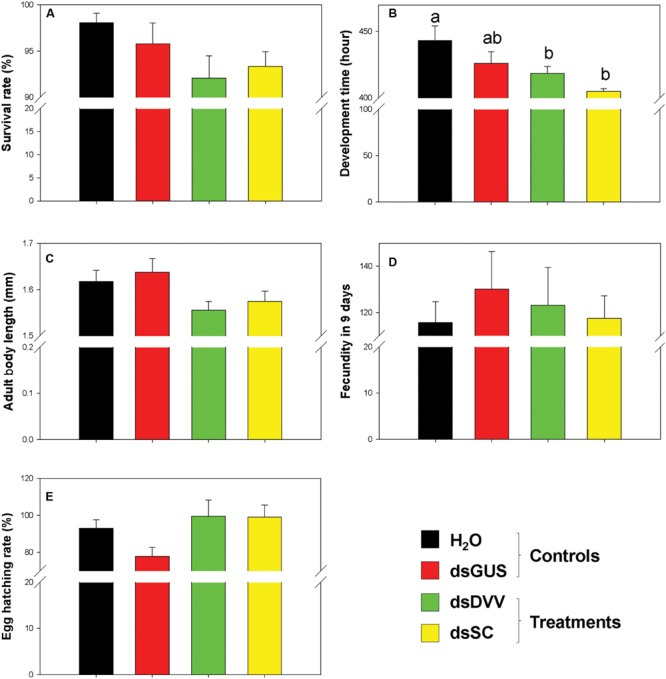
**Phenotypic impacts of dietary RNAi on *S. curviseta*.** The endpoint measurements for *S. curviseta* dietary RNAi toxicity assay include survival rate **(A)**, and life history traits, such as developmental time **(B)**, adult body length **(C)**, fecundity **(D)**, and egg hatching rate **(E)**. See text for treatment details. Values are mean ± SE.

Relative to the control (100% survival rate), all neonate larvae that fed on the diets containing potassium arsenate were dead within eight assay days. There were no significant differences in adult survival rate throughout the treatments (*F*_3,54_ = 2.040, *P* = 0.119; **Figure 5A**). However, the development time from neonate to sex maturity was significantly different among treatments (*F*_3,54_ = 4.544, *P* = 0.007). Specifically, larvae developed faster under the treatments of dsDVV and dsSC than the H_2_O control (**Figure 5B**), although the body length of adults was similar across the treatments (*F*_3,54_ = 2.367, *P* = 0.081; **Figure 5C**). Moreover, fecundity (*F*_3,54_ = 0.248, *P* = 0.863) and hatching rate (*F*_3,49_ = 2.476, *P* = 0.072) were not significantly different across the treatments as well (**Figures 5D,E**).

## Discussion

Hazard and exposure are the two focal points for hypothesis-driven risk assessment. In this study, our focus is the potential hazard of *in planta* RNAi, a newly developed transgenic trait to control insect pests. During the early tier testing, surrogate species were subjected to the laboratory-based, worst-case exposure scenarios, to identify the taxa of concern. The resultant non-target species will then go through the higher tier testing under more realistic semi-field or field conditions.

### The Worst-Case Exposure Scenario

At present, there is limited information on the dose and duration of exposure needed to trigger non-target effects. Given these limitations, it will be difficult to determine the range of dose and the duration of bioassay. Tier I assessments are carried out under a worst-case scenario (US EPA suggest a margin of exposure factor of 10-fold) with purified active ingredients in artificial diets (USEPA, 1998, 2014). In this study, 1.65 μg of artificial diet was fed to 10 individuals every other day for the duration of 28 days, therefore, the average consumption of dsRNA for each *S. curversta* was 2.31 μg [(0.83 μg/μg × 1.65 × 14)/10]. This is equivalent to an exposure of 231 times higher than the LC_50_ reported for *D. v. virgifera* larvae (Baum et al., 2007). For *S. curversta*, the most likely route of exposure to plant-expressed dsRNAs is through the ingestion of pollen, dislodged maize during the planting season, and post-harvest maize residuals deposited in soil. The reported maximum expected environmental concentration of *DvSnf7* dsRNA in MON 87411 pollen, leaf, senescent root, forage root, stover, silk was 0.224, 33.8, 3.68, 4.61, 1.04, 9.02 ng/g fw, respectively (Monsanto, 2013; Tan et al., 2016). In comparison, the estimated margin of exposure to non-target *S. curversta* in this study was 10313-, 68-, 628-, 501-, 2221-, 256-fold, respectively, higher than the documented expression level in the newly deregulated RNAi maize.

To create the worst possible scenario for the non-target impacts, we also intentionally selected a *v-ATPase A* region, representing the highest sequence similarity among target and non-target species, to synthesize arthropod-active dsRNAs. Bachman et al. (2013) investigated the insecticidal spectrum and non-target impacts of a 240 bp dsRNA targeting *D. v. virgifera Snf7*. Authors concluded that phylogenetic relatedness to the target insect pest, and the presence of, at least, one 21-mer (≥21 nt contiguous sequence) match were required for RNAi effects. In this study, dsSC and dsDVV share 85% nucleotide sequence similarity, more importantly, they have multiple 19-23-mer matches. In addition, the insecticidal activity of dsDVV has been confirmed in a previous report (Vélez et al., 2016). When fed with 1 μg of dsDVV per beetle, *D. v. virgifera* adults reached 100% mortality within nine assay days (10% in controls).

### Dietary RNAi Toxicity Assay

Yang et al. (2015) assessed the lethal and sublethal effects of insecticidal *B. thuringiensis* Cry toxins on *F. candida* (Yang et al., 2015), a common soil collembolan sharing the same habitat with *S. curversta*. When fed with diets containing a range of potassium arsenate, *F. candida* exhibited a dose-dependent decline in their survival and growth in comparison to the untreated controls. By the end of the 28-days assay period, the survival rate at the highest concentration (36 ng/μg) was below 40%. In this study, the concentration of potassium arsenate was much higher (100 ng/μl), and led to 100% mortality by the 8th assay day. Potassium arsenate toxicity assay, together with dsRNA stability assay, confirmed the validity of this dietary delivery system for the detection of adverse effects caused by the ingestion of arthropod-active compounds.

Early tier testing, including both temporal RNAi effect study and dietary RNAi toxicity assay, did not observe any treatment effect of ingested dsRNAs on *S. curversta*. Challenges facing the RNAi efficacy in insects include gut pH, diet composition, delivery methods, dose, the timing and duration of exposure, dsRNA structure, length, and concentration, dsRNA uptake and degradation, conservation and function of RNA receptors and transmembrane channels, activation of RNAi machinery, and activity of RNases in digestive fluids and hemolymph (Price and Gatehouse, 2008; Terenius et al., 2011; Scott et al., 2013; Christiaens and Smagghe, 2014). Moreover, previous studies showed that the spectrum of dsRNA activity is expected to be narrow and taxonomically related to the target organism (Baum et al., 2007; Whyard et al., 2009; Bachman et al., 2013). Although dsDVV and dsSC share multiple 21-mer matches, we did not observe apparent adverse effects at both suborganismal and organismal level, suggesting that *in silico* phylogenetic analysis can complement the existing *in vivo* toxicity assay. It is very likely that dsRNA structure is associated with varying efficiency in insects.

Although, the risk assessment framework for RNAi is similar to the one used to assess the risks of *B. thuringiensis* crops and synthetic pesticides, there is a crucial difference between these technologies that pertains to the MOA of siRNAs. For the chemical or microbial pesticides and *B. thuringiensis* crops, the MOA, in general, are well-understood. None of the previous studies have shown any adverse impacts of *B. thuringiensis* toxins to non-target collembolan (Yu et al., 1997; Al-Deeb et al., 2003; Clark and Coats, 2006; Bai et al., 2010, 2011; Römbke et al., 2010; Bakonyi et al., 2011; Yuan et al., 2013; Yang et al., 2015). Without a clear understanding of the MOA of *in planta* RNAi, it is germane to include both survival and life history traits as the measurement endpoints to document both acute and chronic or sublethal effects to the test organisms (Bolognesi et al., 2012; Lundgren and Duan, 2013; Roberts et al., 2015; Xu L.H. et al., 2015). In this study, although there is no apparent adverse effects, *S. curvirseta* larvae indeed developed significantly faster after they ingested dsDVV and dsSC. What does this mean to the test animals? We do not know yet, however, knowing the upstream location where RNAs reside within the central dogma, the inclusion of life history parameters in the endpoint measurements will allow us to monitor the chronic or sublethal effects of RNAi crops on non-target organisms.

In summary, consumption of arthropod-active dsRNAs does not lead to changes in gene expression or adverse effects in the survival and life history traits of *S. curvirseta*, suggesting that the impact of GE RNAi crops on this non-target soil decomposer is negligible. This study develops a standardized dietary RNAi toxicity assay system and provides guidance for the ecological risk assessment (ERA) of RNAi crops in collembolans. Additionally, the publication of negative results will help us document the susceptibility of specific organisms to this new GE trait and contribute to our overall understanding of what non-target arthropods have the potential to be affected by the use of RNAi crops in the environment (Roberts et al., 2015; Xu L.H. et al., 2015). As more information, especially the genomic resources, from different surrogate non-target arthropods that provide diverse ecosystem services becomes available, the possible risks of *in planta* RNAi will be better understood thereby generating an adequate risk assessment framework.

## Materials and Methods

### Insect Culture

The colony of *S. curviseta* Brook (Collembola: Entomobryidae) was kindly provided by Kacie Athey (University of Kentucky). *S. curviseta* cultures were provisioned with russet potato and maintained in plastic containers with a plaster of Paris-charcoal substrate (80 g Plaster of Paris ++ 10 g Charcoal +70 ml dH_2_O; 5 cm D × 4 cm H). Culture containers were covered with foil at 23 ± 0.5°C and 100% relative humidity.

### Molecular Cloning of *v-ATPase subunit A*

The *D. v. virgifera v-ATPase-A* sequence was obtained from a *de novo* transcriptome derived from eggs, neonates and midguts of the third instar larvae (Eyun et al., 2014). To clone *S. curviseta v-ATPase-A*, total RNA was extracted from 20 *S. curviseta* adults using TRIzol (Invitrogen, Carlsbad, CA, USA) following the manufacturer’s instruction. First-strand cDNA was synthesized from 1.0 μg of total RNA using the M-MLV reverse transcription kit (Invitrogen, Carlsbad, CA, USA) according the manufacturer’s recommendations. Degenerate primers were designed based on the conserved *v-ATPase-A* sequences extracted from other insect species. SMARTer^®^ RACE 5′/3′ Kit [TaKaRa Biotechnology (Dalian) Co., Ltd] was used to extend the full length cDNA of *S. curviseta v-ATPase A* following the manufacturer’s protocol. Amplicons were purified and cloned into the pCR4-TOPO vector (Invitrogen, Carlsbad, CA, USA) for sequence confirmation (**Table 1**).

**Table 1:** Primers used in this study.

Primers name	Sequence (5′–3′)^∗^
**dsRNA synthesis**
dsSC F	TAATACGACTCACTATAGGGAGAACACTATTTCCATGTGTTCAG
dsSC R	TAATACGACTCACTATAGGGAGAGCATCTCAGCCAATCG
dsDVV F	TAATACGACTCACTATAGGGAGAGCTCTTTTCCCATGTGTAC
dsDVV R	TAATACGACTCACTATAGGGAGAGCATTTCAGCCAAACG
dsGUS F	TAATACGACTCACTATAGGGAGAGGGCGAACAGTTCCTGATTA
dsGUS R	TAATACGACTCACTATAGGGAGAGGCACAGCACATCAAAGAGA
**RT-qPCR**
*v-ATPase A* RT-qPCR F	TGTCTGGATCTGCTATG
*v-ATPase A* RT-qPCR F	CTTGGTGCGTGATAAAG
*28S* RT-qPCR F	CACGAGTCAGTCGATCCTAAAC
*28S* RT-qPCR R	ACCAGATTCCCTTTCACCTTATC
**Gene cloning**
Degenerate primer F	AGATGTCCGGATCNGCTATGTACGA
Degenerate primer R	ACGAGCAGCCACAGGCATGTT
5′ RACE	
Universal Primer A Mix	CTAATACGACTCACTATAGGGCAAGCAGTGGTATCAACGCAGAGT
*v-ATPase A* R	GACGGTCTTGTAGAAGGGACAGAA
3′ RACE	
Universal Primer A Mix	CTAATACGACTCACTATAGGGCAAGCAGTGGTATCAACGCAGAGT
*v-ATPase A* 1F	GGCCAAATCAATTTACATTCC
*v-ATPase A* 2F	ATATGTCGCTGAAGCTGGAAGTTAC
**Full sequence verification**	
Primer F	GTGCTCGGTTGAAGTGGGATTGAA
Primer R	ATGTACGAGTTGGTTCGTGTCGGT

*^∗^T7 = TAATACGACTCACTATAGGG.*

### Target Region Selection and Bioinformatics Analysis

A 400 nt *v-ATPase-A* dsRNA was designed based on the region of highest sequence similarity among different non-target species including: the honey bee, *Apis mellifera*, the convergent lady beetle, *Hippodamia convergens*, the monarch butterfly, *Danaus plexippus*, and the collembolans, *F. candida* and *S. curviseta*, as well as the western corn rootworm, *D. v. virgifera*. Pairwise sequence alignment was conducted between *D. v. virgifera* and each of the surrogate species via MUSCLE (Edgar, 2004). An in-house Perl script was used to determine the number of Nmer (e.g., 21-mer) in each pairwise alignment. The script searches for any instances of N continuous positions where there are no gaps in any sequences in the alignment. The Nmer sequence as well as the start and end positions of *D. v. virgifera* and *S. curviseta* are illustrated in Supplementary Table S2.

### Phylogenetic Analysis

Phylogenetic tree was reconstructed using MrBayes v3.2.3 (Ronquist et al., 2012) and *v-ATPase A* sequences from *S. curviseta* and 18 insects (Supplementary Table S1). Amino acid sequences were aligned using the MAFFT algorithm in the TranslatorX online platform (Abascal et al., 2010), and the best-fit substitution model was selected by ProtTest 2.4 (Abascal et al., 2005). For MrBayes analysis, the MCMC sampling was run for 2 million generations, sampling trees every 1,000 generations, with the WAG+G model, and the first 25% discarded as burn-in.

### Dietary RNAi Toxicity Assay

To develop a reliable i*n vivo* dietary RNAi toxicity assay, artificial diets were used to deliver arthropod-active compounds to the surrogate organisms. As a positive control for the dietary exposure, potassium arsenate (KH_2_AsO_4_), an arsenic compound, was incorporated into the artificial diet for a toxicity assay. Also, to ensure the uptake, the temporal stability of arthropod-active dsRNAs was examined.

#### Diet Preparation

The artificial diet was prepared according to Giordano et al. (2010) with modifications. Specifically, 0.1 g agar and 1.0 g yeast were dissolved in 5 ml distilled water, respectively. Then, both mixtures were heated to 80°C, maintained for 10 min, and mixed together. Prior to solidification, 40 μl dsRNA solutions (5 μg/μl) were incorporated into a 200 μl yeast-agar mixtures. The final concentration of dsRNA in the artificial diet was 0.83 μg/μl. When the diet cooled, it was poured into a 48-well plate and stored at 4°C. In the preceding phase of the research, 10 individuals, in average, consumed approximately 1.65 μg of artificial diet in 2 days. To ensure the complete uptake of dsRNA and to avoid fungal contamination, the diet cube was chopped into small pieces, and approximately 1.65 μg artificial diet was provided in each container on a small piece of glassine and renewed every other day.

#### Potassium Arsenate Toxicity Assay

Potassium arsenate (Sigma-Aldrich, St. Louis, MO, USA), a known inorganic stomach toxin, was used to test whether the diet is appropriate to be used in the subsequent dietary RNAi toxicity assay. Potassium arsenate was incorporated into the artificial diet as described above, and the final concentration was 100 ng/μl. Potassium arsenate toxicity assay was initially designed to run the same length (28 days) with dietary RNAi toxicity assay. Three replicates were used for this experiment with 10 10-days-old neonate larvae for each replicate.

#### dsRNA Synthesis

Specific primers containing a T7 promoter sequence to generate dsRNAs, including dsSC, dsDVV, and dsGUS, are provided in **Table 1**. The β*-glucuronidase* (GUS) gene was cloned into pBTA2 vector and PCR amplified using gene specific primers, resulting a 560 bp fragment containing a T7 polymerase promoter region at the 5′ end (**Table 1**). PCR amplifications were performed in 50 μl reactions containing 10 μl 5× PCR Buffer (Mg^2+^ Plus), 1.0 μl dNTP mix (10 mM of each nucleotide), 5.0 μl of each primer (10 μM each), and 0.25 μl of GoTaq (5 u/μl; Promega, Madison, WI, USA). The PCR parameters were as follows: one cycle of 94°C for 3 min; 35 cycles of 94°C for 30 s, 59°C for 45 s, and 72°C for 1 min; a final cycle of 72°C for 10 min. The PCR product was used as template to generate dsRNA with the T7MEGAscript kit (Ambion, Austin, TX, USA) following manufacturer’s protocol. The synthesized dsRNAs were suspended in nuclease-free H_2_O, quantified with a NanoDrop 2000c spectrophotometer and then stored at -20°C.

#### dsRNA Stability Assay

To determine the stability of dsRNAs incorporated into the artificial diet in the presence and absence of *S. curviseta*, pieces of diet (3 mm^3^) were placed in plaster of paris-charcoal microcosms using a wax paper. A fraction of diet pieces was harvested in 12 h intervals across two assay days (0, 12, 24, 36, and 48 h). The resultant artificial diets were transferred to 1.5 ml microcentrifuge tubes containing 100 μl nuclease free H_2_O and properly labeled. Using a micropestel, each diet piece was homogenized and resuspended to liberate dsRNAs. After vortexing and centrifuging at 700 g for 5 min, approximately 50–75 μl supernatant were collected, and was subsequently analyzed on a 1% agarose gel. The stability of dsRNA was assessed by the intensity of gel band, and pixel density for each band at each time point was measured using a Bio-Rad Gel Imager (Bio-Rad, Inc., Hercules, CA, USA).

#### Dietary RNAi Toxicity Assay

Ten-days-old neonate larvae were provisioned with artificial diet containing dsDVV, dsSC, dsGUS, and H_2_O. To get the 10-days-old neonate, adults were supplemented with artificial diets and allowed to lay eggs for 1 day. Once eggs were laid, adults were transferred to a fresh container, and the diet was removed. Upon neonate emergence, fresh yeast-agar diet pieces (1.5 μg) were provisioned and replaced every other day to reduce fungal contamination. At day 10, these larvae were selected and transferred using a fine wet brush to each container. A total of 10 individuals were used in one replicate, and 14–15 replications were conducted for each treatment. Assays were carried out in small containers (diameter = 5 cm, height = 4 cm) with a 2 cm layer of plaster of paris and black charcoal (2:1 by volume, mixed with 15 ml ddH_2_O, solidified, saturated before use). Experiments were conducted at 21 ± 1°C in total darkness and 100% relative humidity.

The survival rate and life history traits, including developmental time, fecundity (number of eggs laid), hatching rate of newly laid eggs, and adult body length were measured. Larvae were fed on artificial diets containing dsRNAs for 28 consecutive days. For each replicate, 1.65 μg diet was fed to 10 individuals every other day, so, the average consumption of dsRNA by each individual for 28 consecutive days was 2.31 μg [calculated by (0.83 μg/μg × 1.65 × 14)/10]. Specifically, the number of adults survived at the end of the 28-days feeding test was recorded and the survival rate was calculated for each replicate. Developmental time from egg hatch to maturity was recorded when the first egg appeared. Before transfer to the second egg-laying container, digital photos were taken under magnification and the body length of mature adults was measured from the anterior margin of the head to the end of the posterior abdominal segment. The adults were transferred to a new container and allowed to lay eggs for 9 days. Adults were then removed and fecundity was calculated (indicated by the number of eggs produced in 9 days from the first appearance of eggs). The hatching rate of the above 9 days produced eggs was recorded in another 9 days after adult removal.

### Temporal Profile of Dietary RNAi in *S. curviseta*

#### Reverse Transcriptase-Quantitative Polymerase Chain Reaction (RT-qPCR)

Reverse transcriptase-quantitative polymerase chain reaction (RT-qPCR) primers for *28S ribosomal RNA* (*28S rRNA*) and *v-ATPaseA* were designed based on the sequences obtained from GenBank (EF192441) and this study, respectively, using a web-based tool, https://sg.idtdna.com/Primerquest/Home/ Index. Total RNA was extracted using TRIzol (Invitrogen, Carlsbad, CA, USA) following the manufacturer’s instruction. First-strand cDNA was synthesized from 1.0 μg of total RNA using the M-MLV reverse transcription kit (Invitrogen, Carlsbad, CA, USA) and a random N primer according the manufacturer’s recommendations.

Gene-specific primers (**Table 1**) were used in PCR reactions (15 μl) containing 5.25 μl of ddH_2_O, 7.5 μl of 2 × SYBR Green MasterMix (Bio-Rad, Hercules, CA, USA), 4 μM of each specific primer, and 1.0 μl of first-strand cDNA template. The RT-qPCR program included an initial denaturation for 3 min at 95°C, followed by 40 cycles of denaturation at 9°C for 10 s, annealing for 30 s at 55°C, and extension for 30 s at 7°C. For melting curve analysis, a dissociation step cycle (5°C for 10 s and then 0.5°C for 10 s until 95°C) was added. Relative expression of v-ATPaseA was normalized to a reference gene, 28s rRNA using the 2^-ΔΔCt^ method (Livak and Schmittgen, 2001). The reactions were set up in 96-well format Microseal PCR plates (Bio-Rad, Hercules, CA, USA) in triplicate. Three biological replicates were conducted for each experiment.

#### Temporal Profile of RNAi Effects in *S. curviseta*

While toxicity assay focuses on the impact of dietary RNAi at the organismal level, including survival rate and life history traits, this study is intended to investigate the suborganismal impact. Following the design of dietary RNAi toxicity assay, *v-ATPaseA* expression was measured across all treatments and controls. *S. curviseta* samples were collected on day 0, 2, 4, and 6 to monitor the temporal changes of *v-ATPaseA* expression when 10-days-old neonate larvae ingested dsRNAs. Samples were snap frozen in liquid nitrogen at each time point, and stored in 1.5 ml microcentrifuge tubes at -80°C.

### Statistical Analysis

A one-way ANOVA was used to compare the survival rate, development time, fecundity, hatching rate and the adult body length across different treatments. A two-way ANOVA was used to compare the gene expression dynamics of *v-ATPase A* under different treatments and time. Due to a non-normal distribution of datasets, the non-parametric Kruskal–Wallis test was adopted to analyze the average percent pixel intensity of the gel band for the dsRNA stability assay. Means were compared with LSD tests at *P* < 0.05. SPSS version 20.0 (SPSS, Inc., Chicago, IL, USA) was used for statistical analyses.

## Author Contributions

XZ, BS designed the experiment. HP, LX, HL, JN performed the experiment. XZ contributed reagents/materials. HP, HL analyzed the data. HP, JN, BS, XZ wrote the paper.

## Conflict of Interest Statement

The authors declare that the research was conducted in the absence of any commercial or financial relationships that could be construed as a potential conflict of interest.

## Supplementary Material

FIGURE S1 **Alignment of *v-ATPase A* ORFs between *Sinella curviseta* and *Diabrotica virgifera virgifera*.** Identical nucleotides are highlighted in black boxes.

FIGURE S2 **Standard curves of *v-ATPase A* and *28S rRNA* for RT-qPCR analysis**.

FIGURE S3 **Expression profile of *28S rRNA* across all experimental conditions**.
