# Supplementary figures and images for: Assessment of Potential Risks of Dietary RNAi to a Soil Micro-arthropod, Sinella curviseta Brook (Collembola: Entomobryidae)
Source: Front Plant Sci. 2016 Jul 15;7:1028. doi: 10.3389/fpls.2016.01028 (PMC4945638; doi:10.3389/fpls.2016.01028)

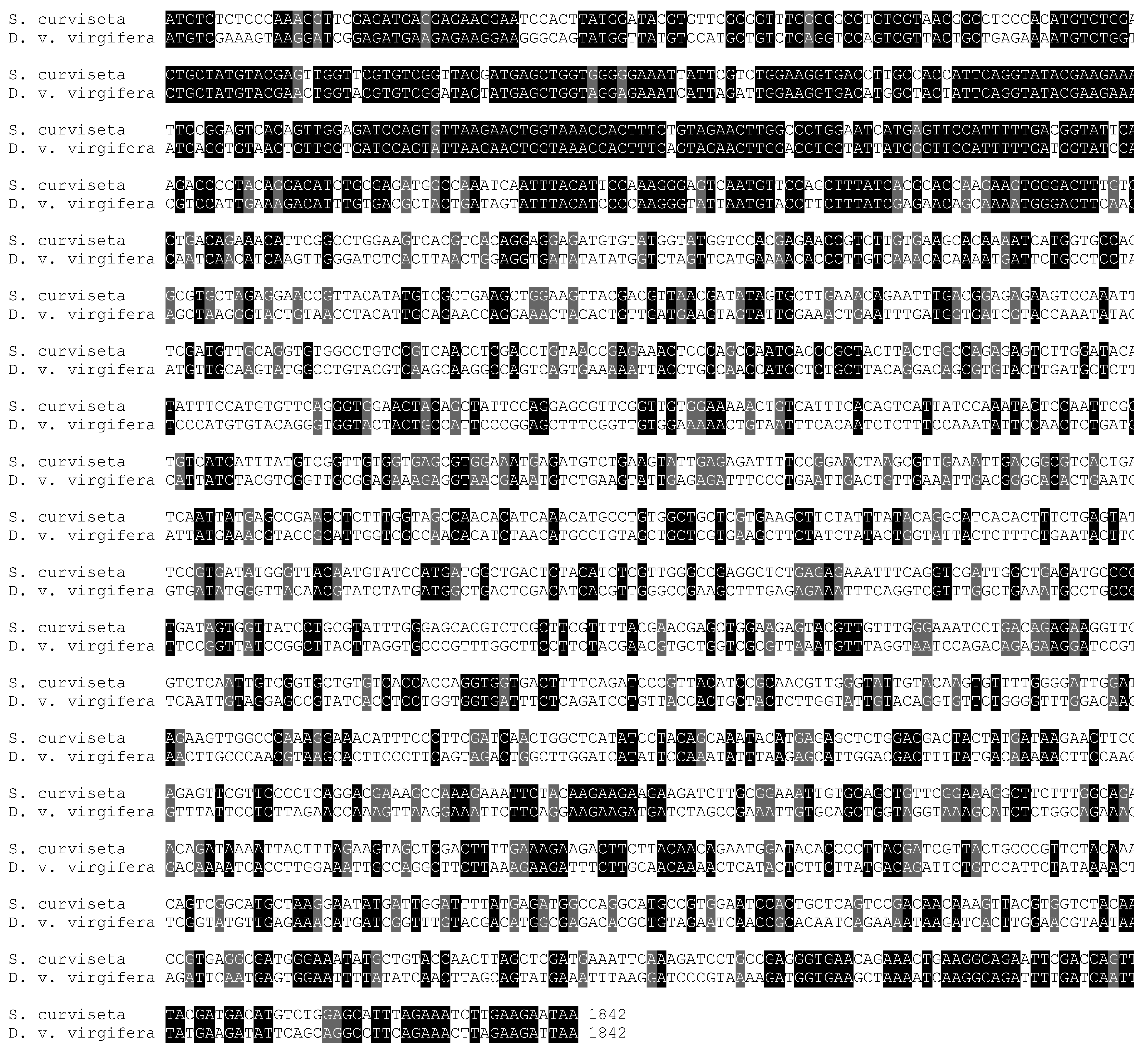

Supplement: FIGURE S1 — Alignment of v-ATPase A ORFs between Sinella curviseta and Diabrotica virgifera virgifera. Identical nucleotides are highlighted in black boxes. [file Image_1.TIFF]

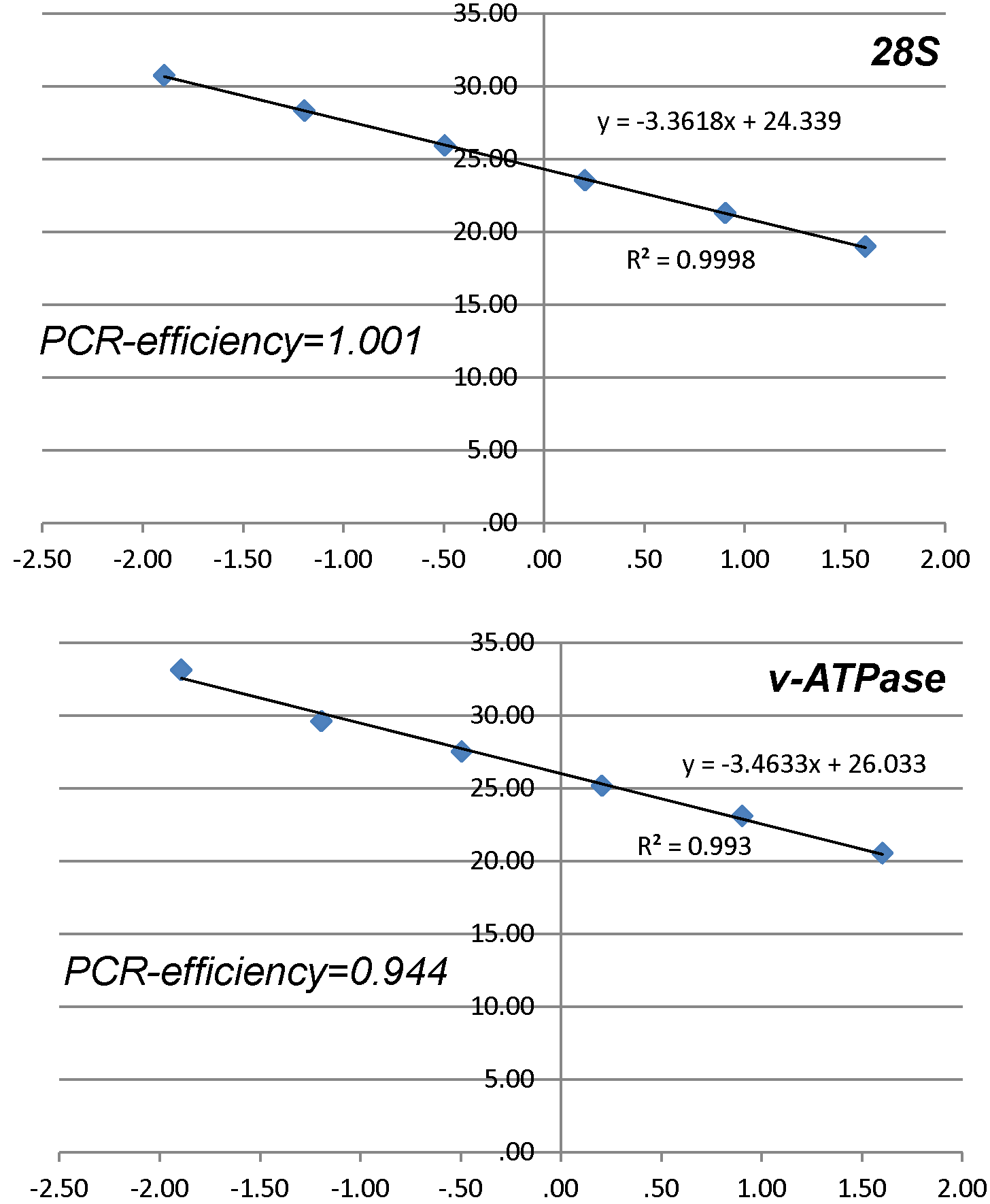

Supplement: FIGURE S2 — Standard curves of v-ATPase A and 28S rRNA for RT-qPCR analysis. [file Image_2.TIFF]

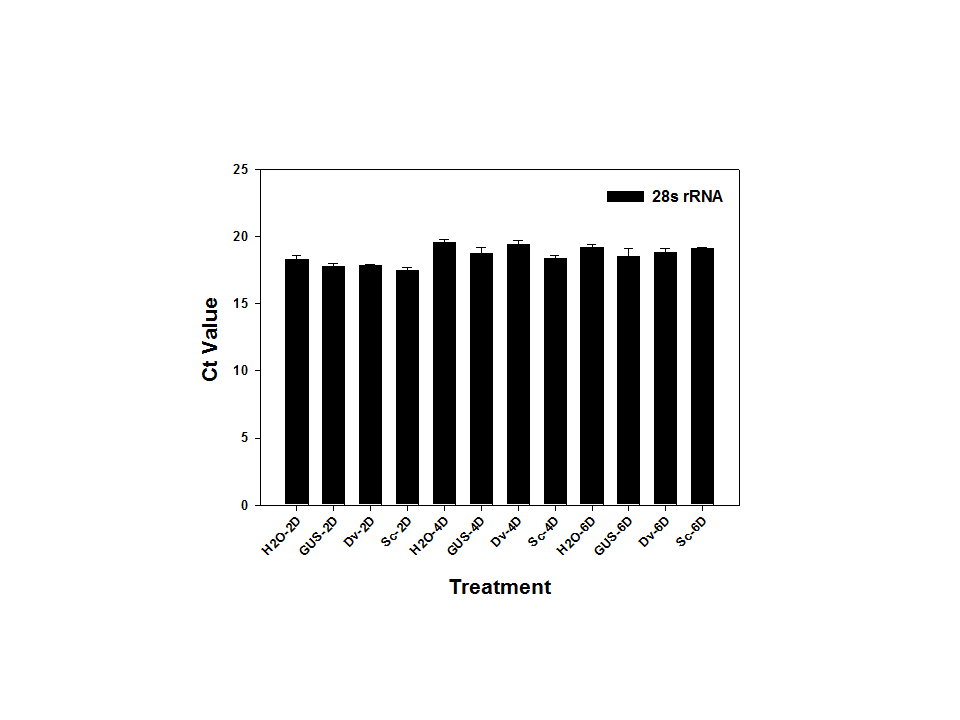

Supplement: FIGURE S3 — Expression profile of 28S rRNA across all experimental conditions. [file Image_3.TIF]
