# Supplementary material for: Assessment of Potential Risks of Dietary RNAi to a Soil Micro-arthropod, Sinella curviseta Brook (Collembola: Entomobryidae)
Source: Front Plant Sci. 2016 Jul 15;7:1028. doi: 10.3389/fpls.2016.01028 (PMC4945638; doi:10.3389/fpls.2016.01028)
Supplement: Supplementary file 4 [file Table_1.DOCX]

**Table S1. Insect information used for the phylogenetic analysis**

| Species | GenBank No. |
| --- | --- |
| *Nasonia vitripennis* | XM_008208806 |
| *Apis mellifera* | XM_006567413 |
| *Bombus impatiens* | XM_003492267 |
| *Tribolium castaneum* | XM_971095 |
| *Plutella xylostella* | XM_011563933 |
| *Bombyx mori* | NM_001098359 |
| *Ostrinia furnacalis* | FR727328 |
| *Aedes albopictus* | AY864912 |
| *Musca domestica* | XM_005179917 |
| *Drosophila ananassae* | XM_001962829 |
| *Acyrthosiphon pisum* | XM_008181186 |
| *Diaphorina citri* | XM_008471983 |
| *Reticulitermes flavipes* | KC569741 |
| *Riptortus pedestris* | AK417332 |
| *Planococcus citri* | JX443529 |
| *Metaseiulus occidentalis* | XM_003741031 |
