# Supplementary material for: Assessment of Potential Risks of Dietary RNAi to a Soil Micro-arthropod, Sinella curviseta Brook (Collembola: Entomobryidae)
Source: Front Plant Sci. 2016 Jul 15;7:1028. doi: 10.3389/fpls.2016.01028 (PMC4945638; doi:10.3389/fpls.2016.01028)
Supplement: Supplementary file 5 [file Table_2.DOCX]

**Table S2. The number of Nmer in each pair-wise alignment between *S. curviseta* and D. v. virgifera *v-ATPase* *A.***

| Matched Sequence* | Length | Query  Start** | Query End | Subject Start*** | Subject End |
| --- | --- | --- | --- | --- | --- |
| ATTCAGGTATACGAAGAAA | 19 | 308 | 326 | 413 | 431 |
| TTCAGGTATACGAAGAAAC | 19 | 309 | 327 | 414 | 432 |
| TTAAGAACTGGTAAACCAC | 19 | 356 | 374 | 461 | 479 |
| TAAGAACTGGTAAACCACT | 19 | 357 | 375 | 462 | 480 |
| AAGAACTGGTAAACCACTT | 19 | 358 | 376 | 463 | 481 |
| AGAACTGGTAAACCACTTT | 19 | 359 | 377 | 464 | 482 |
| GAACTGGTAAACCACTTTC | 19 | 360 | 378 | 465 | 483 |
| TTCGGTTGTGGAAAAACTG | 19 | 869 | 887 | 977 | 995 |
| TCGGTTGTGGAAAAACTGT | 19 | 870 | 888 | 978 | 996 |
| ATGTCTGAAGTATTGAGAG | 19 | 965 | 983 | 1073 | 1091 |
| TGTCTGAAGTATTGAGAGA | 19 | 966 | 984 | 1074 | 1092 |
| GTCTGAAGTATTGAGAGAT | 19 | 967 | 985 | 1075 | 1093 |
| TCTGAAGTATTGAGAGATT | 19 | 968 | 986 | 1076 | 1094 |
| CTGAAGTATTGAGAGATTT | 19 | 969 | 987 | 1077 | 1095 |
| GCTGCTCGTGAAGCTTCTA | 19 | 1076 | 1094 | 1184 | 1202 |
| CTGCTCGTGAAGCTTCTAT | 19 | 1077 | 1095 | 1185 | 1203 |
| TTCCGTGATATGGGTTACA | 19 | 1124 | 1142 | 1232 | 1250 |
| TCCGTGATATGGGTTACAA | 19 | 1125 | 1143 | 1233 | 1251 |
| TGAGAGAAATTTCAGGTCG | 19 | 1188 | 1206 | 1296 | 1314 |
| ATTCAGGTATACGAAGAAAC | 20 | 308 | 327 | 413 | 432 |
| TTAAGAACTGGTAAACCACT | 20 | 356 | 375 | 461 | 480 |
| TAAGAACTGGTAAACCACTT | 20 | 357 | 376 | 462 | 481 |
| AAGAACTGGTAAACCACTTT | 20 | 358 | 377 | 463 | 482 |
| AGAACTGGTAAACCACTTTC | 20 | 359 | 378 | 464 | 483 |
| TTCGGTTGTGGAAAAACTGT | 20 | 869 | 888 | 977 | 996 |
| ATGTCTGAAGTATTGAGAGA | 20 | 965 | 984 | 1073 | 1092 |
| TGTCTGAAGTATTGAGAGAT | 20 | 966 | 985 | 1074 | 1093 |
| GTCTGAAGTATTGAGAGATT | 20 | 967 | 986 | 1075 | 1094 |
| TCTGAAGTATTGAGAGATTT | 20 | 968 | 987 | 1076 | 1095 |
| GCTGCTCGTGAAGCTTCTAT | 20 | 1076 | 1095 | 1184 | 1203 |
| TTCCGTGATATGGGTTACAA | 20 | 1124 | 1143 | 1232 | 1251 |
| TTAAGAACTGGTAAACCACTT | 21 | 356 | 376 | 461 | 481 |
| TAAGAACTGGTAAACCACTTT | 21 | 357 | 377 | 462 | 482 |
| AAGAACTGGTAAACCACTTTC | 21 | 358 | 378 | 463 | 483 |
| ATGTCTGAAGTATTGAGAGAT | 21 | 965 | 985 | 1073 | 1093 |
| TGTCTGAAGTATTGAGAGATT | 21 | 966 | 986 | 1074 | 1094 |
| GTCTGAAGTATTGAGAGATTT | 21 | 967 | 987 | 1075 | 1095 |
| TTAAGAACTGGTAAACCACTTT | 22 | 356 | 377 | 461 | 482 |
| TAAGAACTGGTAAACCACTTTC | 22 | 357 | 378 | 462 | 483 |
| ATGTCTGAAGTATTGAGAGATT | 22 | 965 | 986 | 1073 | 1094 |
| TGTCTGAAGTATTGAGAGATTT | 22 | 966 | 987 | 1074 | 1095 |
| TTAAGAACTGGTAAACCACTTTC | 23 | 356 | 378 | 461 | 483 |
| ATGTCTGAAGTATTGAGAGATTT | 23 | 965 | 987 | 1073 | 1095 |

“*”: Matches within the conserved 400 bp fragments are highlighted in grey.

“**”: Query is the *D. v. vugifera* *v-ATPase subunit A* sequence

“***”: Subject is the *S. curviseta* *v-ATPase subunit A* sequence
